# Neoagarooligosaccharide Protects against Hepatic Fibrosis via Inhibition of TGF-β/Smad Signaling Pathway

**DOI:** 10.3390/ijms22042041

**Published:** 2021-02-18

**Authors:** Ji Hye Yang, Sae Kwang Ku, IL Je Cho, Je Hyeon Lee, Chang-Su Na, Sung Hwan Ki

**Affiliations:** 1 College of Korean Medicine, Dongshin University, Naju, Jeollanam-do 58245, Korea; uranus2k@nate.com; 2 College of Korean Medicine, Daegu Haany University, Gyeongsan, Gyeongsangbuk-do 38610, Korea; gucci200@hanmail.net (S.K.K.); skek023@dhu.ac.kr (I.J.C.); 3 Dyne Bio Inc. Seongnam-si, Gyeonggi-do 13209, Korea; jhl@dynebio.co.kr; 4 College of Pharmacy, Chosun University, Seoseok-dong, Gwangju 61452, Korea

**Keywords:** neoagarooligosaccharides, hepatic stellate cells, liver fibrosis, TGF-β, smad

## Abstract

Hepatic fibrosis occurs when liver tissue becomes scarred from repetitive liver injury and inflammatory responses; it can progress to cirrhosis and eventually to hepatocellular carcinoma. Previously, we reported that neoagarooligosaccharides (NAOs), produced by the hydrolysis of agar by β-agarases, have hepatoprotective effects against acetaminophen overdose-induced acute liver injury. However, the effect of NAOs on chronic liver injury, including hepatic fibrosis, has not yet been elucidated. Therefore, we examined whether NAOs protect against fibrogenesis in vitro and in vivo. NAOs ameliorated PAI-1, α-SMA, CTGF and fibronectin protein expression and decreased mRNA levels of fibrogenic genes in TGF-β-treated LX-2 cells. Furthermore, downstream of TGF-β, the Smad signaling pathway was inhibited by NAOs in LX-2 cells. Treatment with NAOs diminished the severity of hepatic injury, as evidenced by reduction in serum alanine aminotransferase and aspartate aminotransferase levels, in carbon tetrachloride (CCl_4_)-induced liver fibrosis mouse models. Moreover, NAOs markedly blocked histopathological changes and collagen accumulation, as shown by H&E and Sirius red staining, respectively. Finally, NAOs antagonized the CCl_4_-induced upregulation of the protein and mRNA levels of fibrogenic genes in the liver. In conclusion, our findings suggest that NAOs may be a promising candidate for the prevention and treatment of chronic liver injury via inhibition of the TGF-β/Smad signaling pathway.

## 1. Introduction

Liver fibrosis is a highly conserved wound healing response to chronic liver injury [1]. Similar to other tissues, such as the skin, the liver accumulates excessive extracellular matrix (ECM), especially type I and III collagens, as well as proteoglycans, fibronectin, elastin and laminin following injury. In most cases, liver fibrosis is the result of viral and metabolic liver diseases [2,3]. Liver fibrosis may progress to advanced stages of irreversible liver diseases, such as liver cirrhosis and hepatocellular cancer, over time. Currently, liver transplantation is the only treatment available for patients with advanced liver fibrosis. Therefore, new antifibrotic therapies are needed to prevent the late stages of decompensated chronic liver diseases. 

During progression of hepatic fibrosis, activation of hepatic stellate cells (HSCs) plays an important role in the excessive production of ECM proteins. Quiescent HSCs store 80% of total liver retinoids (vitamin A) as well as triglycerides, cholesteryl esters, cholesterol, phospholipids and free fatty acids in lipid droplets in the cytoplasm [4]. In contrast, in injured livers, activated HSCs transform into myofibroblasts, with simultaneous loss of their lipid droplets and production of ECM, subsequently leading to hepatic fibrosis. Among various pathogenic factors, transforming growth factor-β (TGF-β) is a key mediator of liver fibrosis via HSC activation. TGF-β forms receptor complexes, with type I receptors in combination with type II receptors. This leads to the activation of kinase domains within the ligand-bound receptors, which triggers phosphorylation cascades involving Smad transcription factors. Activation of Smads regulates the expression of profibrotic genes, including collagens [5], plasminogen activator inhibitor-1 (PAI-1) [6], proteoglycans [7], integrins [8], connective tissue growth factor (CTGF) [9] and matrix metalloproteases (MMPs) [10]. Inhibition of the TGF-β/Smad signaling pathway attenuates the progression of liver fibrosis in vitro and in vivo [11,12].

β-agarase specifically cleaves the β-1,4 glycosidic bond of agarose to produce neoagarooligosaccharides (NAOs)—which have various degrees of polymerization, in contrast to β-agarase—and cleaves the β-1,3 linkage in agarose to produce agarooligosaccharides. Previously, we reported that NAOs inhibit metabolic liver disease and acute liver injury in mice administered a high cholesterol diet or acetaminophen overdose, respectively [13,14]. Moreover, NAOs present no signs of toxicity up to 5,000 mg/kg body weight/day in acute, repeated 14-day and 91-day oral toxicity tests [15]. These results strongly support the potential application of NAOs in dietary supplements and medications. Nevertheless, the therapeutic efficacy and mechanism of NAOs in chronic liver injury have not yet been elucidated. 

In this study, we show that NAOs attenuate hepatic fibrosis both in vitro and in vivo. NAOs antagonized TGF-β-induced profibrotic gene expression through inhibition of the Smad signaling pathway in cultured immortalized human semi-activated HSCs. Moreover, NAOs reduced the progression of carbon tetrachloride(CCl_4_)-induced liver fibrosis as evidenced by serum liver enzymes, histopathological changes, and production of ECM proteins. Therefore, our results indicate that NAOs have hepatoprotective effects in TGF-β-treated HSCs and CCl_4_-induced liver fibrosis, and have potential applications in clinical interventions. 

## 2. Results

### 2.1. Suppressive Effect of NAOs on HSCs Activation in Vitro

**Figure 1:**
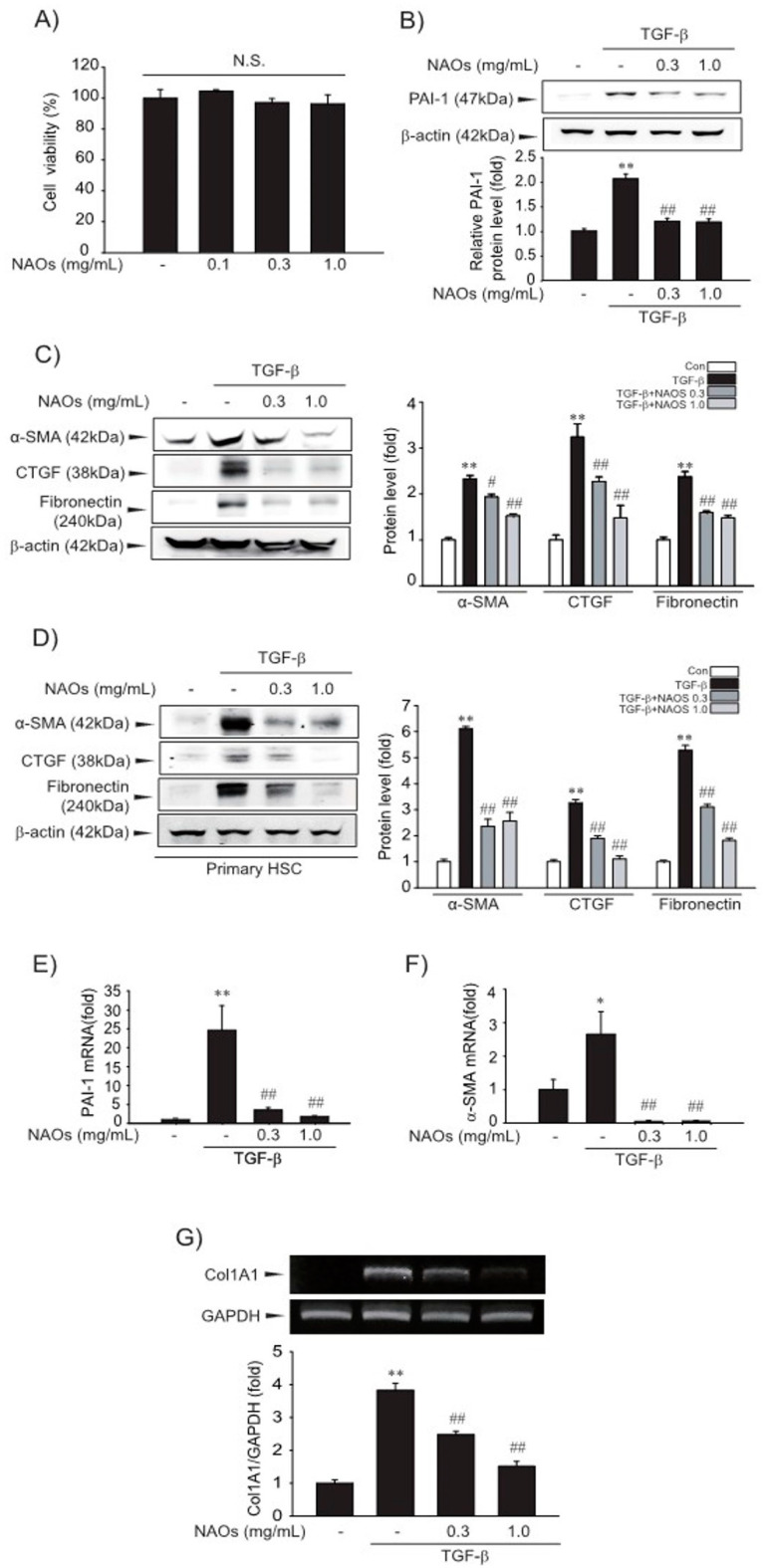
Inhibition of TGF-β-mediated fibrosis markers by neoagarooligosaccharides (NAOs). (**A**) MTT assays for cell viability. Effect of NAOs (0.1–1.0 mg/mL, 12 h treatment) on cell viability of LX-2 cells, a well characterized human HSC line evaluated using MTT assays (*n* = 3). N.S., not significant. (**B**–**D**) Effect of varying concentrations of NAOs on TGF-β-mediated fibrogenic protein expression. Cells were treated with 0.3–1.0 mg/mL NAOs and incubated with TGF-β (1 ng/mL) for 6 h. Protein levels in the cell lysates were determined by immunoblotting (*n* = 3). (**E**,**F**) Real-time PCRanalysis. Cells were treated with 0.3–1.0 mg/mL NAOs for 30 min, and then further incubated with TGF-β for 3 h. The α-SMA and PAI-1 transcripts were analyzed by Real-time PCR, with the mRNA level of *GAPDH* used as a housekeeping gene (*n* = 3). (**G**) Collagen (Col) 1A1 transcripts analyzed by RT-PCR assays. Results were confirmed by repeated experiments (*n* = 3). Data represent the mean ± standard error (S.E.) of three replicates; ** *p* < 0.01, * *p* < 0.05, vs. vehicle-treated control; ^##^ *p* < 0.01, ^#^ *p* < 0.05, vs. TGF-β alone. Minus sign (-) indicates untreated with NAOs.

First, we examined the cytotoxicity of NAOs in LX-2 cells by MTT assay. We found that there were no significant differences between vehicle- and NAOs-treated cells at concentrations up to 1.0 mg/mL (Figure 1A). Therefore, we adopted 0.3–1.0 mg/mL NAOs in following experiments, to demonstrate the effect of NAOs on HSC activation. Treatment of NAOs in LX-2 cells effectively inhibited TGF-β-induced PAI-1, α-SMA, CTGF and fibronectin protein expression—all of which are typical markers of HSC activation (Figure 1B–C, Figures S1 and S2). When we used isolated primary HSC from mice, we also observed increased PAI-1, α-SMA, CTGF and fibronectin expression; TGF- was antagonized by NAOs (Figure 1D). RT-PCR analysis confirmed that TGF-β markedly increased mRNA levels of PAI-1, α-SMA and collagen 1A1 (COL1A1), which were almost completely blocked by pretreatment with NAOs (Figure 1E–G). These findings indicate that NAOs inhibit HSC activation. 

### 2.2. Inhibitory Effect of NAOs on TGF-β/Smad Signaling 

**Figure 2:**
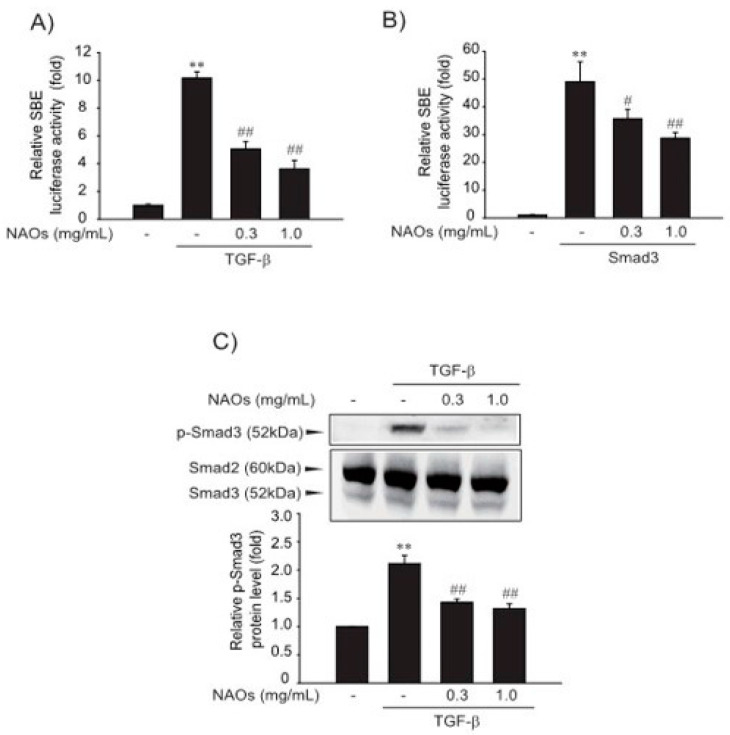
Inhibition of TGF-β downstream signaling by neoagarooligosaccharides (NAOs). (**A**) Effect of NAOs on TGF-β-induced Smad pathway in the LX-2 cells, determined by transfecting the cells with pGL-SBE-luciferase construct, followed by treatment with TGF-β (1 ng/mL) and/or NAOs. (**B**) LX-2 cells co-transfected with Smad3 expression construct and treated with NAOs (12 h). (**C**) Immunoblotting for Smad3 phosphorylation. Cells treated with 0.3–1.0 mg/mL NAOs for 30 min prior to incubation with TGF-β for 30 min. Cell lysates immunoblotted for phosphorylated Smad3 and results confirmed by repeating the experiments. Data represent the mean ± S.E. of three replicates; ** *p* < 0.01, vs. vehicle-treated control; ^##^ *p* < 0.01, ^#^ *p* < 0.05, vs. TGF-β alone. Minus sign (-) indicates untreated with NAOs.

Next, we investigated the effect of NAOs on the TGF-β-induced Smad pathway, a major mediator of TGF-β signaling. First, to verify the underlying molecular mechanism of inhibition of HSC activation, we performed reporter gene assay of Smad-binding element (SBE)-luciferase activity, which contained nine repeated SBEs. SBE-luciferase activity was significantly increased by TGF-β; however, pretreatment with NAOs (0.3 or 1.0 mg/mL) inhibited SBE-reporter gene activity in LX-2 cells (Figure 2A). Furthermore, Smad3-dependent transcription of SBE reporter activity was inhibited by pretreatment with NAOs (Figure 2B). Consistent with these findings, the increase in TGF-β-induced Smad3 phosphorylation was antagonized by pretreatment with NAOs (Figure 2C). These findings suggest that NAOs inhibit the TGF-β/Smad signaling pathway.

### 2.3. Suppression of CCl_4_-Induced Liver Fibrosis by NAOs In Vivo

**Figure 3:**
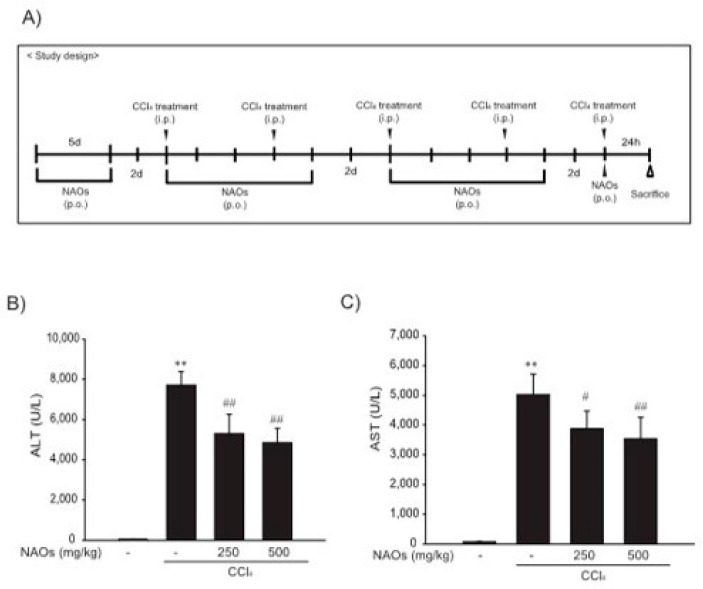
Inhibition of CCl_4_-induced liver injury by neoagarooligosaccharides (NAOs). (**A**) Study design. Prior to CCl_4_ treatment to induce liver fibrosis, mice are treated with NAOs for 5 days. Liver fibrosis is then induced by intraperitoneal injection of CCl_4_ (0.5 mg/kg, dissolved in olive oil [10%]) into mice, twice a week for 2 weeks. Mice are also treated with NAOs five times a week for two weeks. Twenty-four hours prior to sacrifice, mice are induced with a final intraperitoneal injection of CCl_4_. (**B**,**C**) Activities of serum alanine aminotransferase (ALT) and aspartate aminotransferase (AST) analyzed using an automated blood chemistry analyzer. All values are expressed as mean ± S.E. of five mouse serum samples (** *p* < 0.01, vs. vehicle control; ^##^ *p* < 0.01, ^#^ *p* < 0.05, significant vs. CCl_4_).

**Figure 4:**
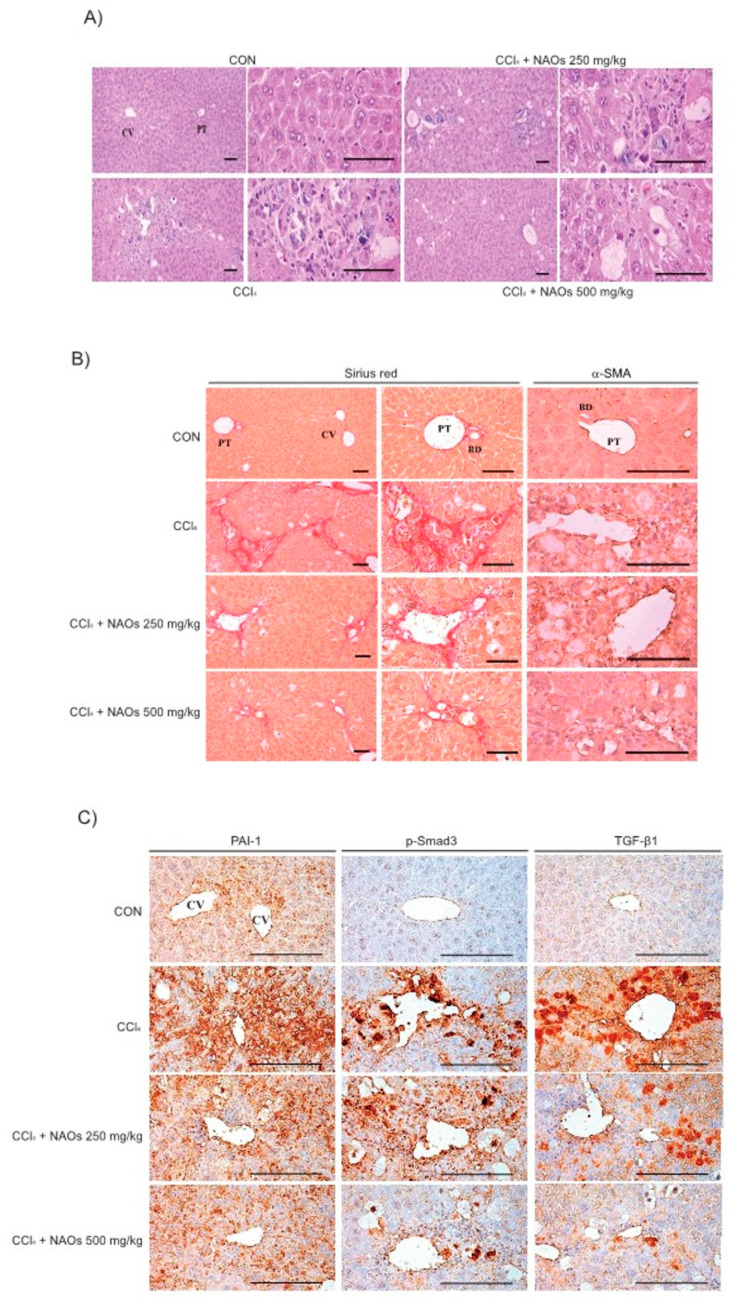
Inhibition of CCl_4_-induced liver fibrosis by neoagarooligosaccharides (NAOs). (**A**) Histopathological changes in mice with CCl_4_-induced subacute liver damage, showing centrilobular necrosis, including ballooning of hepatocytes and infiltration of inflammatory cells. CCl_4_+NAOs treated mice show markedly lowered CCl_4_-induced subacute liver damage as compared to CCl_4_-treated mice. CCl_4_: Carbon tetrachloride, CV: Central vein, PT: Portal trial, Scale bar = 120 μm. (**B**,**C**) Sirius red and α-SMA, PAI-1, p-Smad3 and TGF-β1 immunohistochemical staining of liver from mice treated with CCl_4_ or CCl_4_+NAOs for 2 weeks (Scale bar = 120 μm). CON, vehicle control.

**Figure 5:**
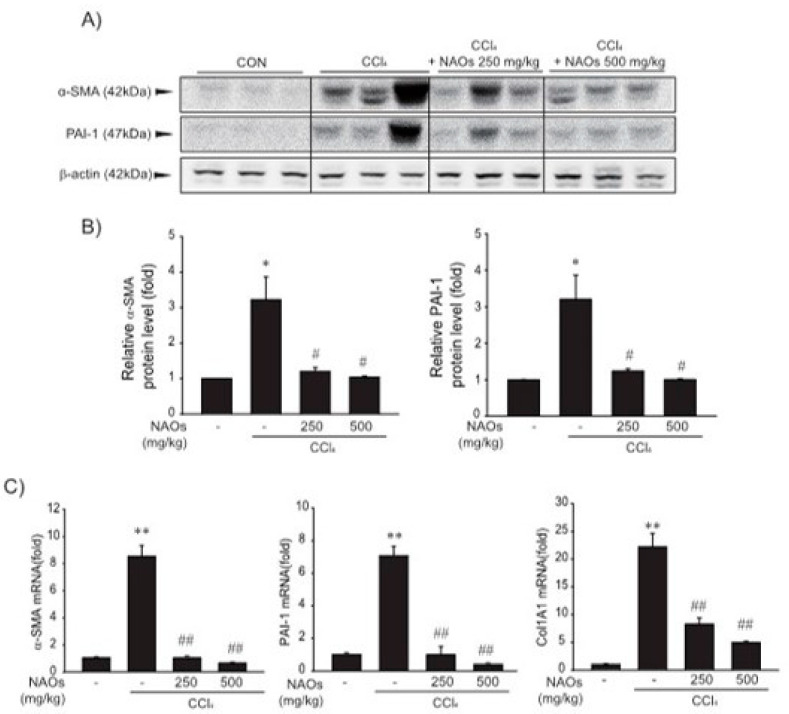
Inhibition of CCl_4_-induced expression of fibrogenic genes by neoagarooligosaccharides (NAOs). (**A**,**B**) Western blot analysis. α-SMA and PAI-1 protein levels assessed by immunoblotting. (*n* = 3). Results are presented as means ± S.E. of three replicates. (**C**) α-SMA, PAI-1, and Col 1A1 transcripts in CCl_4_-induced mice liver assessed by real-time PCR analysis (*n* = 3). Results are presented as means ± S.E.; ** *p* < 0.01, * *p* < 0.05, vs. vehicle control; ^##^ *p* < 0.01, ^#^ *p* < 0.05, vs. CCl_4_; CON, vehicle control.

To study the inhibitory effects of NAOs in vivo, we used the classic animal model of CCl_4_-induced liver fibrosis. This is the most common adopted method of inducing pernicious effects in the liver by producing highly reactive metabolites, resulting in liver lesions and subsequent fibrosis [16,17]. We pretreated mice with NAOs for 5 days before CCl_4_ treatment to induce liver fibrosis. CCl_4_ was administered twice a week for two weeks in concomitant daily treatment with NAOs (Figure 3A). Mice were sacrificed 24 h after the final CCl_4_ administration. First, we analyzed the levels of the serum biomarkers of liver damage, including alanine aminotransferase (ALT) and aspartate aminotransferase (AST). Injection of CCl_4_ for 2 weeks significantly increased serum ALT and AST levels; however, these effects were considerably inhibited by NAOs treatment (Figure 3B,C). To determine the hepatoprotective effects of NAOs against CCl_4_-induced liver damage, we performed histopathological analyses to assess the extent of liver injury. Administration of CCl_4_ led to degenerative regions, liver centrilobular necrosis, and increased numbers of degenerative hepatocytes and inflammatory cells. However, these CCl_4_-induced hepatic damages were markedly reduced by NAOs treatment (Figure 4A; Table 1). We stained the mice liver sections with Sirius red to examine the effect of NAOs on collagen accumulation. The results showed that the CCl_4_-treated mice had large amounts of collagen deposition in the fibrotic septa between nodules. In contrast, treatment with NAOs decreased the collagen accumulation following CCl_4_ administration (Figure 4B, left; Table 1). HSC activation is a major component of liver fibrosis, and α-SMA is a key marker for HSC activation [18]. α-SMA immunoreactive cells were significantly increased in the centrilobular regions following CCl_4_ treatment, but this increase in α-SMA immunoreactive cells was markedly inhibited by treatment with NAOs (Figure 4B, right; Table 1). In addition, treatment of NAOs decreased PAI-1, p-Smad3 and TGF-β1 immunoreactive cells by CCl_4_-treatment (Figure 4C and Table 1). Moreover, the antifibrotic effect of NAOs was confirmed by immunoblotting and real-time PCR analysis of the major markers (PAI-1, α-SMA, and Col1A1) of liver fibrosis taken from three randomly selected mouse samples (Figure 5A–C).

## 3. Discussion

Hepatic fibrosis occurs when healthy liver tissues becomes scarred from chronic liver injury and inflammatory responses, and therefore cannot function normally [19,20]. Chronic liver damage leads to liver fibrosis in conjunction with the accumulation of ECM proteins, which is a typical characteristic of most chronic liver diseases [21]. Activated HSCs are major ECM-producing cells in an injured liver; these cells are activated by fibrogenic mediators, such as TGF-β [22]. TGF-β—a key downstream mediator in liver fibrogenesis—exerts its effect via downstream molecular signaling pathways, such as Smad2 and Smad3 [23]. TGF-β bound type II receptor leads to recruitment and phosphorylation of the type I receptor. Ligand-receptor complex formation leads to the activation of kinase domains within the receptors, which triggers phosphorylation of Smads [24]. The Smads then transmit the signals from the receptors of the TGF-β superfamily members to the nucleus, where they initiate transcription of TGF-β target genes [25]. In the present study, treatment with NAOs inhibited TGF-β-induced profibrogenic gene expression in LX-2 cells. Moreover, we found that NAOs blocked the Smad signaling pathway downstream of TGF-β. NAOs might therefore act as an antagonist of TGF-β receptors. 

Next, we evaluated the protective effect of NAOs against CCl_4_-induced hepatic fibrosis in vivo. CCl_4_, a well-known fibrosis-inducing hepatotoxin, is extensively used in liver-related studies. CCl_4_-treated rodents exhibited fatty changes along with an increase in inflammatory cell infiltrations, damage to normal hepatocytes, deposition of collagen and formation of fiber segmentation [26]. Chronic liver toxicity induced by CCl_4_ causes an increase in lipid peroxidation that leads to a decrease in antioxidant enzymes and glutathione activities [27,28]. This CCl_4_-induced chronic toxicity was suppressed by pretreatment with NAOs. Furthermore, the increase in serum ALT and AST levels induced by administration of CCl_4_ for 2 weeks was decreased by NAOs treatment (Figure 3). NAOs also dramatically inhibited CCl_4_-induced hepatic damage, including regions of degeneration, liver centrilobular necrosis, and the increased number of degenerative hepatocytes and inflammatory cells. Treatment with NAOs also reduced CCl_4_-induced collagen accumulation and synthesis of various ECM components, including PAI-1, α-SMA, and Col1A1 (Figure 4 and Figure 5, Table 1). The in vivo effects of NAOs on liver fibrosis might be due to the protection of hepatocytes and the inhibition of HSC activation. Hepatic regeneration after liver injury is composed not only of fibroblasts, but also of cholangiocytes that orchestrate the deposition of fibrosis by stimulating proliferation and activation of myofibroblasts [29]. Further study of the effect of NAOs on liver regeneration is still required and currently ongoing.

**Figure 6:**
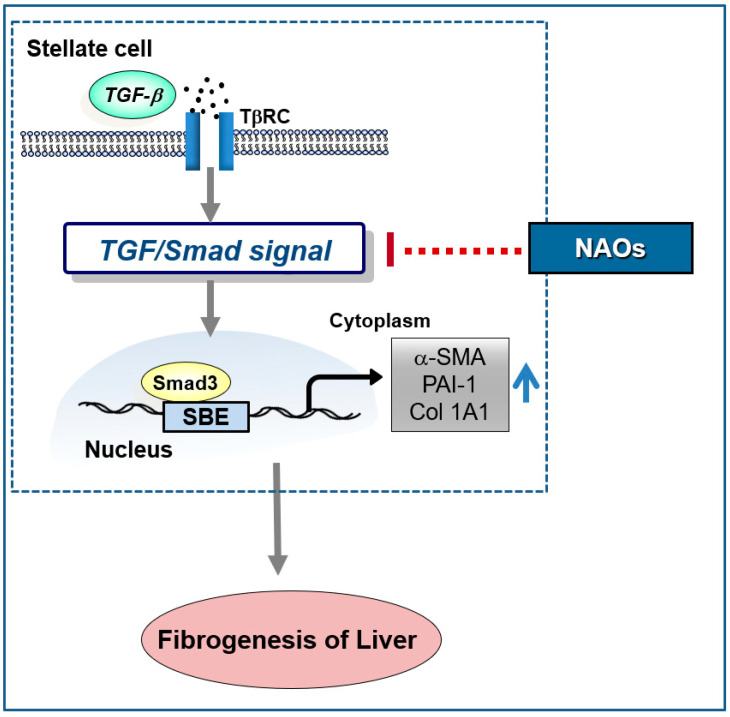
Schematic diagram demonstrating the mechanism through which neoagarooligosaccharides (NAOs) protect against hepatic fibrosis via inhibition of TGF-β/Smad signaling pathway.

In conclusion, our study demonstrates that NAOs prevent liver fibrosis in vitro and in vivo. Furthermore, NAOs significantly protect hepatocytes from injury and inflammation following CCl_4_ administration. Collectively, these findings suggest that NAOs are a potential antifibrotic agent for the prevention and treatment of chronic liver diseases via inhibition of HSC activation (Figure 6).

## 4. Materials and Methods

### 4.1. Materials

PAI-1 (614024) and fibronectin (610077) antibodies were obtained from BD Biosciences (Franklin Lakes, NJ, USA). Phospho-Smad3 (#9520) and Smad2/3 (#3102) antibodies were provided by Cell Signaling (Danvers, MA, USA). CTGF antibodies (SC-101586) were purchased from Santa Cruz Biotechnology (Santa Cruz, CA), and TGF-β1 antibody (NBP1-80289) was obtained from Novus Biologicals (Littleton, CO). Horseradish peroxidase-conjugated goat anti-rabbit (G21234) and anti-mouse (G21040) antibodies were purchased from Invitrogen (Carlsbad, CA, USA). α-SMA (A2547) and β-actin (A5441) antibodies, MTT (M2128), and dimethylsulfoxide (D8418) were acquired from Sigma Chemicals (St. Louis, MO, USA). TGF-β (240-B) was purchased from R&D Systems (Minneapolis, MN, USA). CTGF antibody (sc-101586) obtained from Santa Cruz Biotechnology (Santa Cruz, CA). CCl_4_ (33650-0330) was received from Junsei Chemical (Kyoto, CO).

### 4.2. Preparation of NAOs

Agar from *Gelidium elegans* was hydrolyzed to NAOs by *β-agarase* DagA originating from *Streptomyces coelicolor* A3(2)_M22-2C43. Enzymatic reactions occurred at 44~46 °C for 16 h. NAOs solution was filtered and concentrated to 10 times its concentration. Freeze-dried NAO was homogenized into 100 mesh-sized powders.

### 4.3. Cell Culture

Immortalized human semi-activated HSCs and LX-2 cells were kindly donated by Dr. S. L. Friedmann (Mount Sinai School of Medicine, NY, USA), and maintained in Dulbecco’s modified Eagle’s medium (DMEM), containing 10% fetal bovine serum, 50 units/mL penicillin/streptomycin at 37 °C in a humidified 5% CO_2_ atmosphere. Primary HSCs were isolated from the livers of 8-week-old ICR mice (Oriental Bio, Sungnam, Korea) as previously reported [11,28]. After intubation through the portal vein, the livers were perfused in situ with Ca^2+^-free Hank’s balanced saline solution at 37 °C for 15 min and then perfused with a solution containing 0.05% collagenase and Ca^2+^ for 15 min at a flow rate of 10 mL/min. The perfused livers were minced, filtered through a 70 m cell strainer (BD Biosciences), and centrifuged at 50× *g* for 3 min to separate the supernatant and pellet. The pellet was then discarded. Next, the supernatant was further centrifuged at 500× *g* for 10 min, resuspended in Ficoll plus Percoll (1:10; GE Healthcare, Chicago, IL, USA), and centrifuged at 1400× *g* for 17 min. HSCs were collected from the interface. 

### 4.4. Animals

The animal experiments were conducted in accordance with the National Institutes of Health Guide for the Care and Use of Laboratory Animals and approved by the Institutional Animal Care and Use Committee of Chosun University (Approval No. CIACUC2015-A0043, 22 December 2015). Male ICR mice (six weeks old) were obtained from Oriental Bio (Sung-nam, Korea) and acclimatized for 1 wk. Mice (*n* = 5/group) were housed at 20 ± 2 °C with 12 h light/dark cycle and a relative humidity of 50 ± 5% under filtered, pathogen-free air, with food (Purina, Korea) and water available ad libitum.

### 4.5. CCl_4_-Induced Hepatic Fibrosis

CCl_4_-induced hepatic fibrosis model was established as described previously [30]. To induce liver fibrosis, CCl_4_ dissolved in olive oil (10%) was intraperitoneally injected (0.5 mg/kg) into the mice thrice a week for 2 weeks, and NAOs dissolved in tap water were administered orally for 5 days per week (Figure 3A). The mice were induced by intraperitoneal injection with CCl_4_ 24 h prior to sacrifice.

### 4.6. Blood Chemistry 

Plasma ALT and AST were analyzed using Spectrum^®^, an automatic blood chemistry analyzer (Abbott Laboratories, Abbott Park, IL, USA).

### 4.7. Histological Process

Approximately equal regions of individual hepatic samples were crossly trimmed. All crossly trimmed hepatic tissues were refixed in 10% neutral buffered formalin for 24 h, at least in this histopathological observation. After paraffin embedding, 3–4 μm sections were prepared as three serial sections in each liver in paraffin blocks. Representative sections were stained with hematoxylin and eosin (H&E) for general histopathological profiles [31], Sirius red for collagen fiber [32], or Avidin-biotin-peroxidase complex (ABC)-based immunohistochemistry, against a profibrogenic cytokine involved in hepatic fibrosis–TGF-β (1:100) with its target signal molecule–pSmad3 (1:100), against α-SMA (1:100) and PAI-1 (1:100), according to previously established methods. Two histological fields in each hepatic tissue, totalling 10 histological fields in each group, were considered for further statistical analysis in the present histopathological observation. The percentage of degenerative regions (%/mm^2^) in livers showing centrilobular necrosis, congestion and inflammatory cell infiltrations on hepatic lobules were calculated using a computer-based automated image analyzer (iSolution FL ver 9.1, IMT i-solution Inc., Vancouver, Quebec, Canada) with collagen fiber-occupied region percentages around central veins noted as %/mm^2^ of hepatic parenchyma under Sirius red staining. The cells occupied by over 20% of immunoreactivities, the density of PAI-1, p-Smad3 and TGF-β1 were regarded as positive, and mean numbers of α-SMA, PAI-1, p-Smad3 and TGF-β1 immunopositive cells were calculated as cells/1000 hepatocytes using a computer-based image analyzer and histological camera system (Nikkon, Tokyo, Japan). The histopathologist was also blind to group distribution when this analysis was made.

### 4.8. MTT Assay

To measure cytotoxicity, cells were plated in 96-well plates, treated with the chemicals for 12 or 24 h, and stained with MTT (0.2 mg/mL, 4 h). The medium was then removed from the wells and formazan crystals in the wells were dissolved by adding 200 μL of dimethyl sulfoxide. Absorbance was measured at 540 nm using an enzyme-linked immunosorbent assay microplate reader (Versamax, Molecular Device, Sunnyvale, CA, USA). Cell viability was defined relative to the untreated control (viability [% control] = 100 × [absorbance of treated sample]/[absorbance of control]).

### 4.9. Immunoblot Analysis 

Protein extraction, subcellular fractionation, SDS-polyacrylamide gel electrophoresis and immunoblot analyses were performed as described previously [33]. Briefly, samples were separated by 7.5% or 12% gel electrophoresis and electrophoretically transferred to nitrocellulose membranes. Each nitrocellulose membrane was incubated with the indicated primary antibody (PAI-1, 1:2000; α-SMA, 1:2000; CTGF, 1:200; Phospho-Smad3, 1:1000; Smad2/3, 1:1000 and β-actin, 1:2000) and then with horseradish peroxidase-conjugated secondary antibody (1:500). Immunoreactive proteins were visualized by ECL chemiluminescence (Amersham Biosciences, Buckinghamshire, UK). Equal protein loading was verified using β-actin.

### 4.10. RNA Isolation and Real-Time RT-PCR Analysis 

Total RNA was isolated using TRIzol (Invitrogen, Carlsbad, CA, USA), according to the manufacturer’s instructions. To obtain cDNA, total RNA (2 µg) was reverse-transcribed using oligo(dT)_16_ primer. The cDNA obtained was amplified with a high-capacity cDNA synthesis kit (Bioneer, Daejon, Korea) using a thermal cycler (Bio-Rad, Hercules, CA, USA). Real-time PCR was performed with STEP ONE (Applied Biosystems, Foster City, CA, USA) using SYBR green premix according to the manufacturer’s instructions (Applied Biosystems). Primers were synthesized by Bioneer. The following primer sequences were used: human α-SMA 5’-CGCATCCTCATCCTCCCT-3’ (sense) and 5’-GGCCGTGATCTCCTTCTG-3’ (antisense); human PAI-1 5’-CGCCAGAGCAGGACGAA-3’ (sense) and 5’-CATCTGCATCCTGAAGTTCTCA-3’ (antisense); human Col 1A1 5’-CCTGGGTTTCAGAGACAACTTC-3’ (sense); mouse α-SMA 5’-TCCTCCCTGGAGAAGAGCTAC-3’ (sense) and 5’-TATAGGTGGTTTCGTGGATGC-3’ (antisense); mouse PAI-1 5’-GACACCCTCAGCATGTTCATC-3’ (sense) and 5’-AGGGTTGCACTAAACATGTCAG-3’ (antisense); mouse Col 1A1 5’-ACCTGTGTGTTCCCTACTCA-3’ (sense) and 5’-GACTGTTGCCTTCGCCTCTG-3’ (antisense); and 5’-TCCACATGCTTTATTCCAGCAATC-3’ (antisense). Glyceraldehyde 3-phosphate dehydrogenase (GAPDH) was used as an internal control for RT-PCR. 

### 4.11. Luciferase Gene Assay 

To measure luciferase activity, LX-2 cells were replated in 24-well plates overnight, serum-starved for 6 h, and transiently transfected with SBE-luciferase and pRL-TK plasmids (which encode *Renilla* luciferase and are used to normalize transfection efficacy) in the presence of Lipofectamine^®^ Reagent (Invitrogen, San Diego, CA, USA) for 3 h. Transfected cells were allowed to recover in DMEM for 3 h and then exposed to 1 μg/mL for 12 h. Firefly and *Renilla* luciferase activities in cell lysates were measured using the dual luciferase assay system (Promega) according to the manufacturer’s instructions. Relative luciferase activity was calculated by normalizing firefly luciferase activity to that of *Renilla* luciferase.

### 4.12. Statistical Analysis

One-way analysis of variance (ANOVA) was used to determine the significance of the differences between the treatment groups. The Newman–Keuls test was used to determine the significance of the differences between multiple group means. Results are expressed as mean ± S.E. *p* < 0.05 was considered statistically significant.

## 5. Conclusions

Collectively, the present study clearly shows that NAOs inhibited the expression of profibrotic genes induced by TGF-β in vitro. Furthermore, NAOs antagonized CCl_4_-induced collagen accumulation and synthesis of various ECM components in vivo. Our data strongly indicate that NAOs may be a promising therapeutic candidate to effectively prevent or treat chronic liver diseases via inhibition of HSC activation. 

## Figures and Tables

**Table 1:** Histomorphometrical analysis of hepatic tissues from vehicle or CCl4-treated mice.

Groups Index (Unit)	Controls		CCl_4_ with NAOs	
	Vehicle (*n* = 5)	CCl_4_ (*n* = 5)	250 mg/kg (*n* = 5)	500 mg/kg (*n* = 5)
Hepatic Staging Scores (Max = 6)	0.30 ± 0.48	4.40 ± 0.52 **	3.00 ± 1.05 ^##^	2.10 ± 0.57 ^##^
Degenerative regions (%/mm^2^)	2.08 ± 1.18	54.43 ± 13.37 **	29.61 ± 10.41 ^##^	18.13 ± 10.50 ^##^
Degenerative hepatocytes (cells/1000 hepatocytes)	17.20 ± 11.48	436.70 ± 127.05 **	206.00 ± 109.82 ^##^	126.50 ± 85.64 ^##^
Inflammatory cells (cells/1000 hepatocytes)	24.80 ± 14.37	320.40 ± 89.63 **	168.00 ± 69.00 ^##^	112.00 ± 22.39 ^##^
Sirius red-stained collagen occupied regions (%/mm^2^)	1.75 ± 1.29	29.26 ± 10.69 **	15.13 ± 7.51 ^##^	10.00 ± 4.25 ^##^
Mean α-SMA immunoreactive cell numbers	8.00 ± 5.73	192.40 ± 55.07 **	88.80 ± 28.96 ^##^	48.00 ± 17.02 ^##^
Mean PAI-1 immunoreactive cell numbers	82.40 ± 25.68	660.80 ± 127.15 **	315.20 ± 125.71 ^##^	235.00 ± 113.28 ^##^
Mean p-Smad3 immunoreactive cell numbers	17.60 ± 13.43	340.80 ± 110.16 **	137.60 ± 32.11 ^##^	59.60 ± 22.78 ^##^
Mean TGF-β1 immunoreactive cell numbers	14.20 ± 11.72	375.50 ± 105.59 **	159.40 ± 48.34 ^##^	73.80 ± 26.72 ^##^

Results are presented as mean ± S.D. of ten histological fields; ** *p* < 0.01, vs. vehicle control; ^##^ *p* < 0.01, vs. CCl_4._

## Data Availability

The data presented in this study are available on request from the corresponding author.

## Supplementary Materials

The following are available online at https://www.mdpi.com/1422-0067/22/4/2041/s1.

## Abbreviations

α-SMA	smooth muscle actin
ALT	Aminotransferase
AST	Aspartate aminotransferase
CCl_4_	Carbon tetrachloride
CTGF	Connective tissue growth factor
ECM	Extracellular matrix
HSCs	Hepatic Stellate Cells
NAOs	Neoagarooligosaccharides
PAI-1	Plasminogen activator inhibitor-1
SBEs	Smad-binding elements
TGF-β	Transforming growth factor-β
