# Supplementary figures and images for: Neoagarooligosaccharide Protects against Hepatic Fibrosis via Inhibition of TGF-β/Smad Signaling Pathway
Source: Int J Mol Sci. 2021 Feb 18;22(4):2041. doi: 10.3390/ijms22042041 (PMC7922480; doi:10.3390/ijms22042041)

Fig.1B)

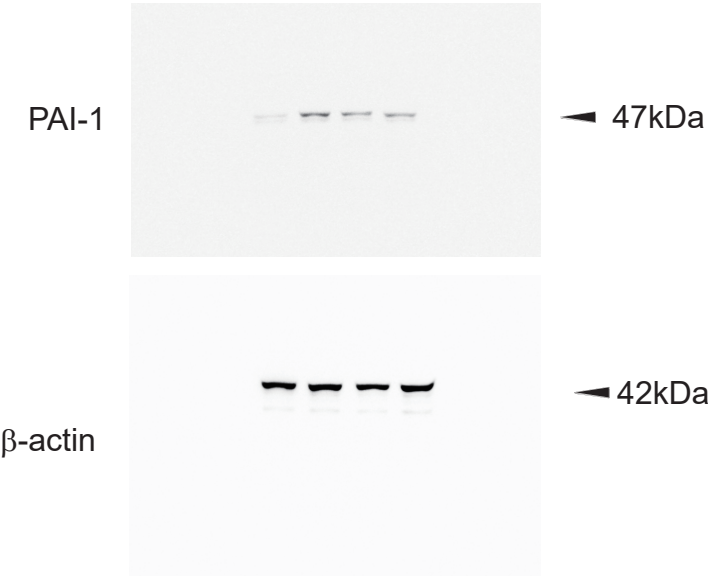

Fig.1D)

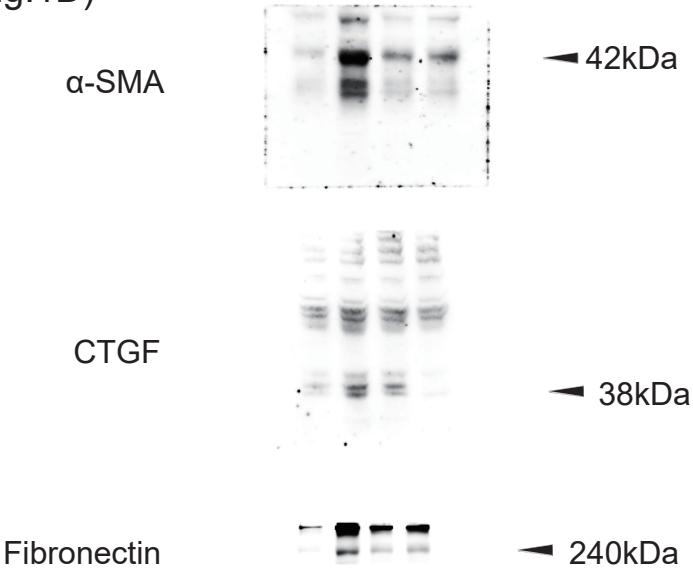

Fig.1C)

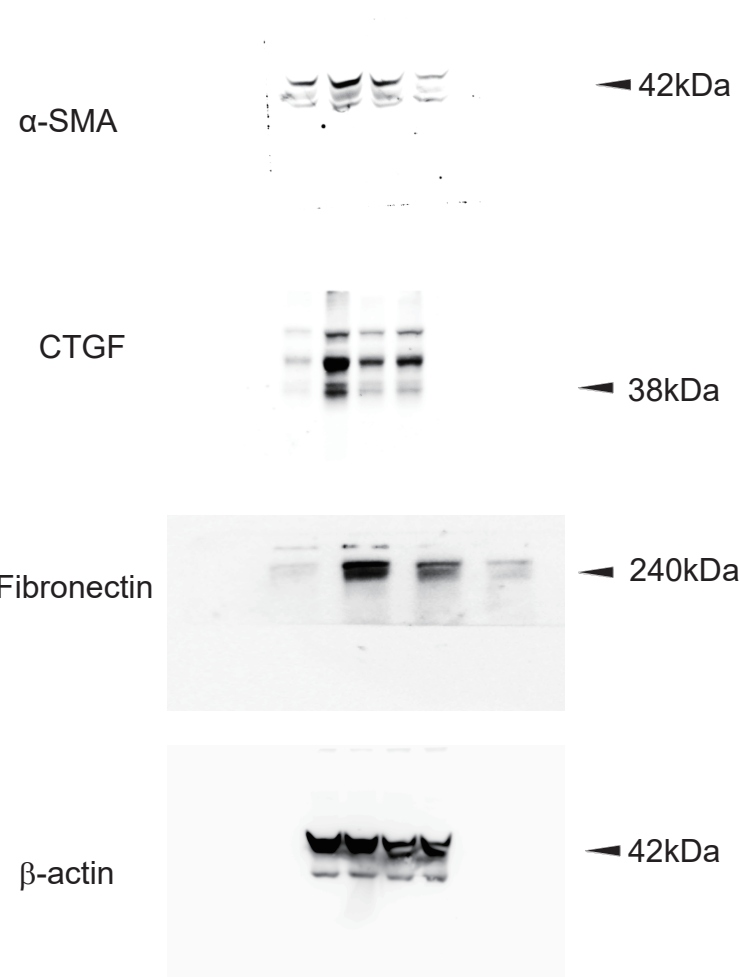

Fig.1H)

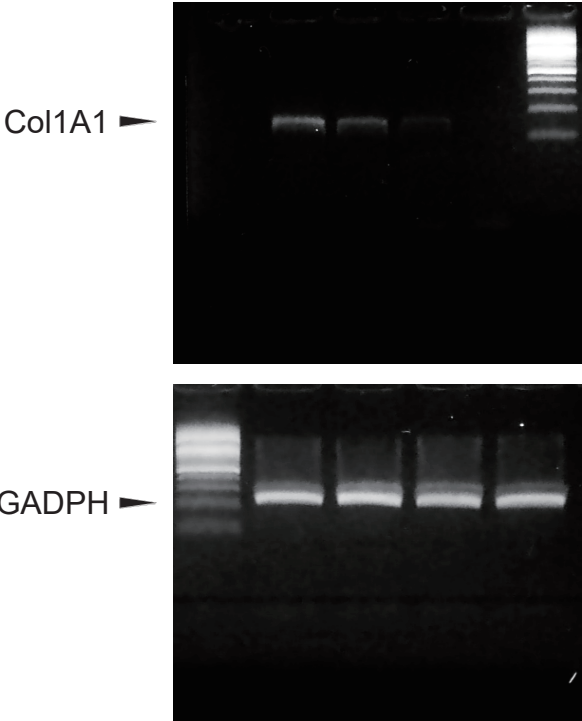

Supplemental Fig 1. (Yang et al.)

Fig. 2C)

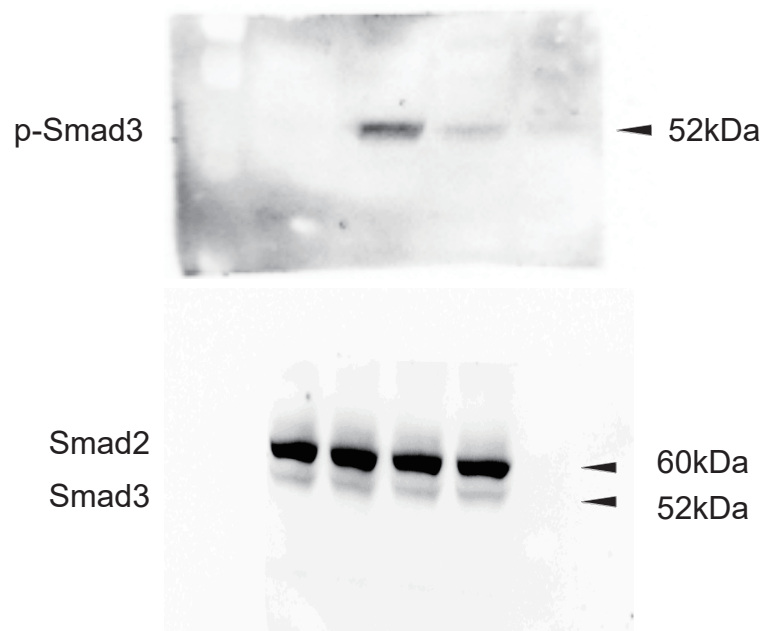

Fig.5A)

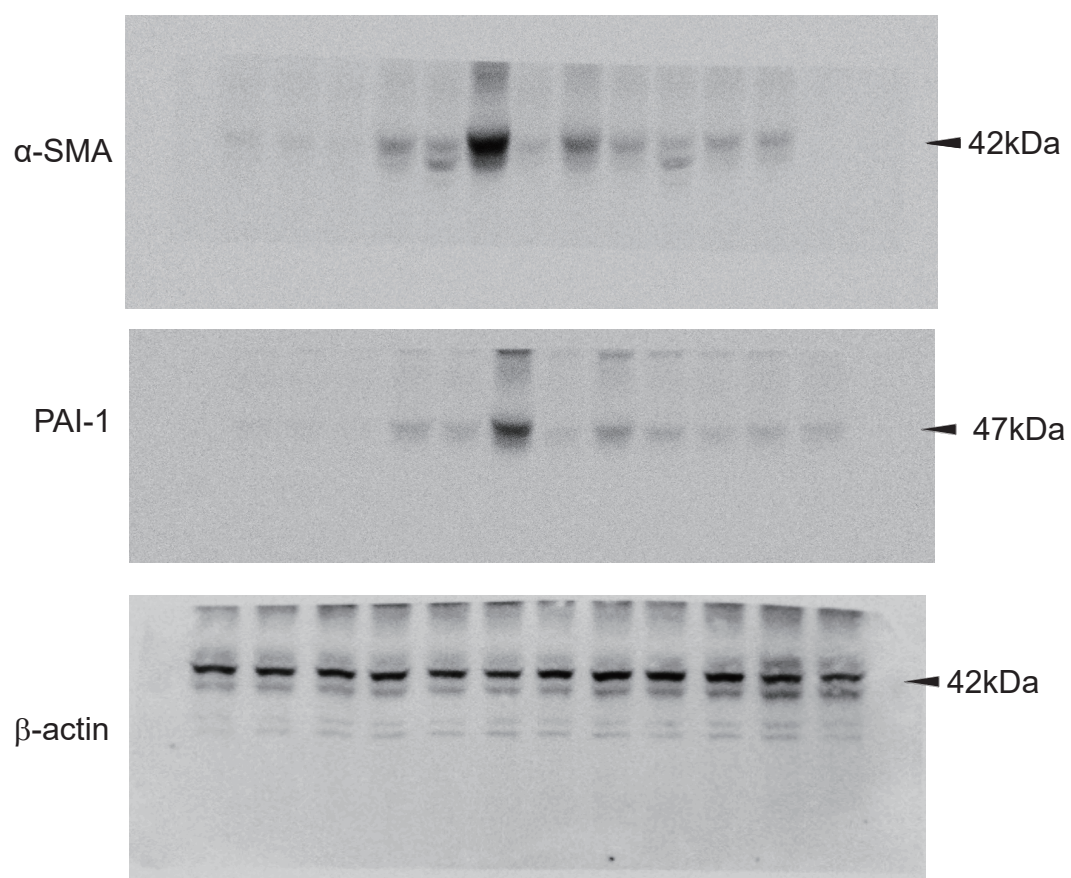

Supplemental Fig 2. (Yang et al.)

Supplement: Supplementary file 1 [file ijms-22-02041-s001.pdf]
